# *N*-[2,6-Bis(1-methyl­eth­yl)phen­yl]pyridine-4-carboxamide

**DOI:** 10.1107/S1600536812038846

**Published:** 2012-09-22

**Authors:** Baptiste Laramée, Mihaela Cibian, Garry S. Hanan

**Affiliations:** a Département de Chimie, Université de Montréal, Pavillon J.-A. Bombardier, 5155 Decelles Avenue, Montréal, Québec, Canada H3T 2B1

## Abstract

In the title compound, C_18_H_22_N_2_O, the dihedral angle between the benzene ring and the pyridine ring is 80.0 (1)°. In the crystal, N—H⋯O hydrogen bonds connect the mol­ecules into chains along the *b* axis. The packing also features C—H⋯O and C—H⋯N hydrogen bonds and C—H⋯π interactions, one directed to the benzene ring and the other to the center of the pyridine ring.

## Related literature

For general review and synthetic details about amide bond generation and application, see: Pattabiraman & Bode (2011 ▶). The title compound has not been reported in coordination chemistry, but complexes of similar ligands are known. For the use of such ligands in coordination chemistry, see: Baytekin *et al.* (2009 ▶); Hasegawa *et al.* (2007 ▶); Kumar *et al.* (2004 ▶). For related benzamide crystal structures, see: Saeed *et al.* (2010 ▶); Zhang & Zhao (2010 ▶); Roopan *et al.* (2009 ▶); Gowda *et al.* (2008 ▶). For background to the synthetic route, see: Boeré *et al.* (1998 ▶); Krajete *et al.* (2004 ▶); Schafer *et al.* (2011 ▶); Wallace *et al.* (1990 ▶). For a description of the Cambridge Structural Database, see: Allen (2002 ▶).
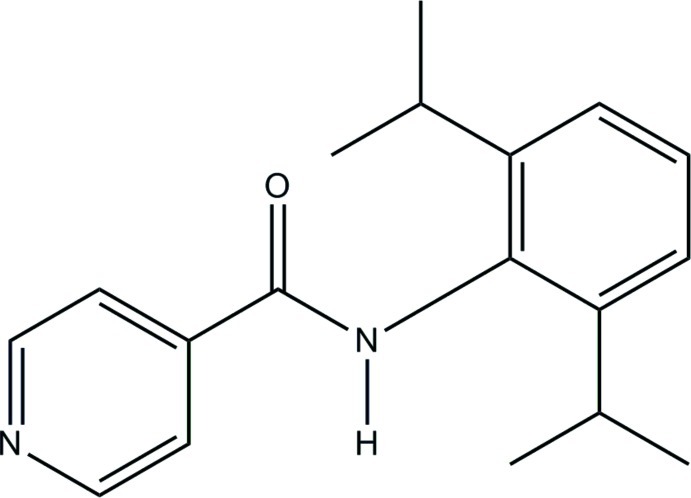


## Experimental

### 

#### Crystal data

C_18_H_22_N_2_O
*M* *_r_* = 282.38
Monoclinic, 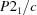

*a* = 9.0994 (1) Å
*b* = 9.8039 (1) Å
*c* = 18.1882 (2) Å
β = 96.8650 (4)°
*V* = 1610.93 (3) Å^3^
*Z* = 4
Cu *K*α radiation
μ = 0.57 mm^−1^
*T* = 150 K
0.20 × 0.12 × 0.12 mm

#### Data collection

Bruker APEXII CCD diffractometer
Absorption correction: multi-scan (*SADABS*; Sheldrick, 1996 ▶) *T*_min_ = 0.895, *T*_max_ = 0.935
81057 measured reflections
3126 independent reflections
3000 reflections with *I* > 2σ(*I*)
*R*_int_ = 0.025

#### Refinement

*R*[*F* ^2^ > 2σ(*F* ^2^)] = 0.036
*wR*(*F* ^2^) = 0.097
*S* = 1.04
3126 reflections
198 parameters
H atoms treated by a mixture of independent and constrained refinement
Δρ_max_ = 0.23 e Å^−3^
Δρ_min_ = −0.17 e Å^−3^

### 

Data collection: *APEX2* (Bruker, 2009 ▶); cell refinement: *SAINT* (Bruker, 2009 ▶); data reduction: *SAINT*; program(s) used to solve structure: *SHELXS97* (Sheldrick, 2008 ▶); program(s) used to refine structure: *SHELXL97* (Sheldrick, 2008 ▶); molecular graphics: *SHELXTL* (Sheldrick, 2008 ▶); software used to prepare material for publication: *UdMX* (Maris, 2004 ▶).

## Supplementary Material

Crystal structure: contains datablock(s) I, global. DOI: 10.1107/S1600536812038846/nk2182sup1.cif

Structure factors: contains datablock(s) I. DOI: 10.1107/S1600536812038846/nk2182Isup2.hkl

Supplementary material file. DOI: 10.1107/S1600536812038846/nk2182Isup3.cml

Additional supplementary materials:  crystallographic information; 3D view; checkCIF report

## Figures and Tables

**Table 1:** Hydrogen-bond geometry (Å, °) *Cg*1 and *Cg*2 are the centroids of the phenyl and pyridyl rings, respectively.

*D*—H⋯*A*	*D*—H	H⋯*A*	*D*⋯*A*	*D*—H⋯*A*
N1—H20⋯O1^i^	0.872 (16)	1.970 (16)	2.7985 (11)	158.3 (13)
C3—H3⋯O1^i^	0.95	2.54	3.4500 (12)	161
C5—H5⋯N2^ii^	0.95	2.61	3.4983 (13)	155
C6—H6⋯*Cg*1^iii^	0.95	2.81	3.6467	150
C15—H15*B*⋯*Cg*2^iv^	0.98	2.97	3.7858	143

Symmetry codes: (i) 
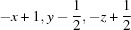
; (ii) 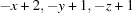; (iii) 
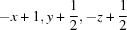
; (iv) 
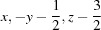
.

**Fig. 1.**
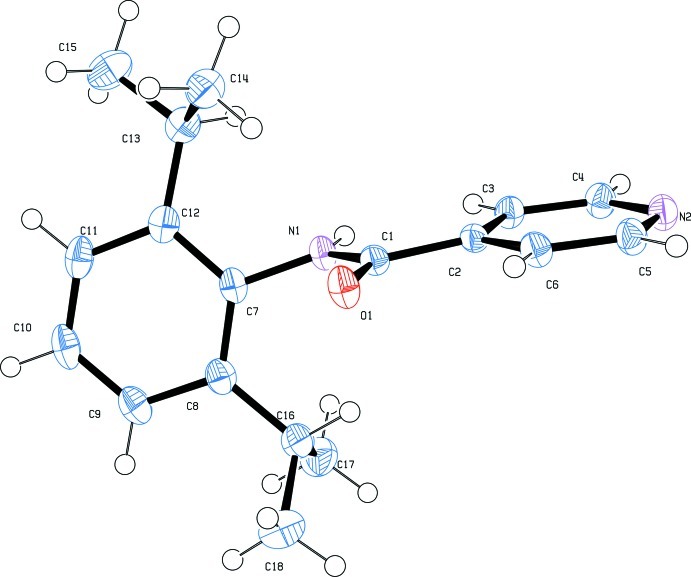
The molecular structure of N-[2,6-bis(1-methylethyl)phenyl]-4-pyridinecarboxamide, with atom labels and displacement ellipsoids drawn at the 50% probability level.

**Fig. 2.**
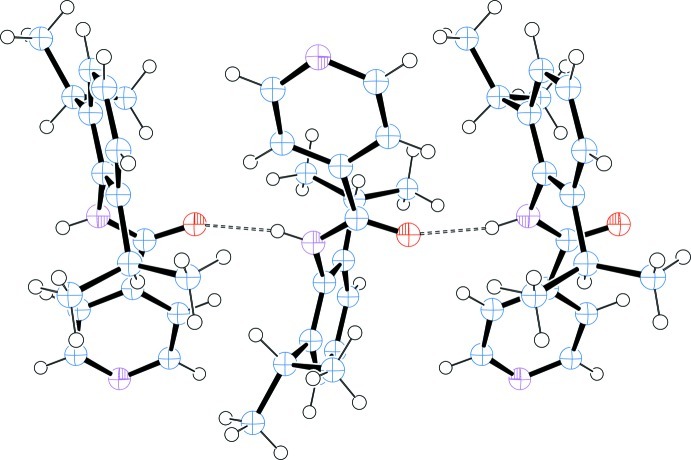
A view of the crystal packing of the title compound, showing one layer of molecules connected by N—H···O hydrogen bonds.

## supplementary crystallographic information

### Comment

In the present work, *N*-[2,6-bis(1-methylethyl)phenyl]-4-pyridinecarboxamide has been synthesized as a potential ligand in coordination chemistry. The metal complexes of similar precursors are already known to catalyse hydroamination of aminoalkenes (Schafer *et al.* (2011)).

The molecular structure of the title compound is illustrated in Fig. 1. The bond distances are normal (Allen (2002)). The pyridyl ring is slightly tilted with respect to the central amide group at the angle of 5.7 (1)°, while the substituted phenyl ring is tilted by 79.8 (1)°.

Fig. 2 illustrates conventional intermolecular N—H···O hydrogen bonding, forming chains along *b* axis.

### Experimental

*N*-[2,6-bis(1-methylethyl)phenyl]-4-pyridinecarboxamide was prepared by combining and modifying reported methods (Wallace *et al.* (1990); Boeré *et al.* (1998); Krajete *et al.* (2004)). Isonicotinic acid (8.00 g, 65.0 mmol) is refluxed in thionyl chloride (30.0 ml), under nitrogen atmosphere, for 3 h. The excess thionyl chloride is distilled off, to afford the corresponding isonicotinoyl chloride (HCl salt) as pale-yellow solid (not isolated). Dry dichloromethane (20.0 ml), dry pyridine (70.0 ml), and 2,6-diisopropylaniline (12.9 ml, 68.2 mmol) are added at 0°C (ice bath). The reaction mixture is left to heat to room temperature, and is subsequently refluxed for 2 h. The green-yellow suspension obtained is reduced to around 70 ml by evaporation under vacuum, and water (150 ml) is added. The green-yellow precipitate formed is recuperated as solid by filtration. Recrystallization from hot methanol at 4°C affords the desired *N*-(2,6-diisopropylphenyl)isonicotinamide as X-ray quality off-white needles, air dried (14.9 g, 52.7 mmol). Yield 81% 1H NMR (300 MHz, DMSO-d6) delta p.p.m. 10.05 (s, 1 H, NH) 8.80 (d, J=6 Hz, 2 H, H-py) 7.89 (d, J=6 Hz, 2 H, H-py) 7.28 - 7.37 (m, 1 H, *p*-H—Ph) 7.22 (d, J=8 Hz, 2 H, *m*-H—Ph) 3.05 (spt, J=7 Hz, 2 H, –CH-(CH3)2) 1.14 (dd, J=18, 7 Hz, 12 H, –CH-(CH3)2)

### Refinement

The amide H atom was located in a difference Fourier map and refined freely. The other H atoms were positioned geometrically (C—H 0.95 Å) and included in the refinement in the riding model approximation; their temperature displacement parameters were set to 1.2 times the equivalent isotropic temperature factors of the parent site.

### Crystal data

**Table:**

C_18_H_22_N_2_O	*F*(000) = 608
*M**_r_* = 282.38	*D*_x_ = 1.164 Mg m^−^^3^
Monoclinic, *P*2_1_/*c*	Cu *K*α radiation, λ = 1.54178 Å
Hall symbol: -P 2ybc	Cell parameters from 9664 reflections
*a* = 9.0994 (1) Å	θ = 4.9–70.9°
*b* = 9.8039 (1) Å	µ = 0.57 mm^−^^1^
*c* = 18.1882 (2) Å	*T* = 150 K
β = 96.8650 (4)°	Block, colourless
*V* = 1610.93 (3) Å^3^	0.20 × 0.12 × 0.12 mm
*Z* = 4	

### Data collection

**Table:**

Bruker APEXII CCD diffractometer	3126 independent reflections
Radiation source: fine-focus sealed tube	3000 reflections with *I* > 2σ(*I*)
Graphite monochromator	*R*_int_ = 0.025
φ and ω scans	θ_max_ = 71.3°, θ_min_ = 4.9°
Absorption correction: multi-scan (*SADABS*; Sheldrick, 1996)	*h* = −11→11
*T*_min_ = 0.895, *T*_max_ = 0.935	*k* = −11→12
81057 measured reflections	*l* = −22→22

### Refinement

**Table:**

Refinement on *F*^2^	Primary atom site location: structure-invariant direct methods
Least-squares matrix: full	Secondary atom site location: difference Fourier map
*R*[*F*^2^ > 2σ(*F*^2^)] = 0.036	Hydrogen site location: inferred from neighbouring sites
*wR*(*F*^2^) = 0.097	H atoms treated by a mixture of independent and constrained refinement
*S* = 1.04	*w* = 1/[σ^2^(*F*_o_^2^) + (0.0527*P*)^2^ + 0.483*P*] where *P* = (*F*_o_^2^ + 2*F*_c_^2^)/3
3126 reflections	(Δ/σ)_max_ < 0.001
198 parameters	Δρ_max_ = 0.23 e Å^−^^3^
0 restraints	Δρ_min_ = −0.17 e Å^−^^3^

### Special details

**Table:**

Experimental. X-ray crystallographic data for I were collected from a single-crystal sample, which was mounted on a loop fiber. Data were collected using a Bruker Platform diffractometer, equipped with a Bruker *SMART* 4 K Charged-Coupled Device (CCD) Area Detector using the program *APEX2* and a Nonius FR591 rotating anode equiped with a Montel 200 optics The crystal-to-detector distance was 5.0 cm, and the data collection was carried out in 512 *x* 512 pixel mode. The initial unit-cell parameters were determined by a least-squares fit of the angular setting of strong reflections, collected by a 10.0 degree scan in 33 frames over four different parts of the reciprocal space (132 frames total). One complete sphere of data was collected, to better than 0.80 Å resolution.
Geometry. All esds (except the esd in the dihedral angle between two l.s. planes) are estimated using the full covariance matrix. The cell esds are taken into account individually in the estimation of esds in distances, angles and torsion angles; correlations between esds in cell parameters are only used when they are defined by crystal symmetry. An approximate (isotropic) treatment of cell esds is used for estimating esds involving l.s. planes.
Refinement. Refinement of *F*^2^ against ALL reflections. The weighted *R*-factor *wR* and goodness of fit *S* are based on *F*^2^, conventional *R*-factors *R* are based on *F*, with *F* set to zero for negative *F*^2^. The threshold expression of *F*^2^ > σ(*F*^2^) is used only for calculating *R*-factors(gt) *etc*. and is not relevant to the choice of reflections for refinement. *R*-factors based on *F*^2^ are statistically about twice as large as those based on *F*, and *R*- factors based on ALL data will be even larger.

### Fractional atomic coordinates and isotropic or equivalent isotropic displacement parameters (Å^2^)

**Table:**

	*x*	*y*	*z*	*U*_iso_*/*U*_eq_	
C1	0.56648 (10)	0.43259 (10)	0.26398 (5)	0.0201 (2)	
C2	0.68103 (10)	0.39496 (10)	0.32743 (5)	0.0195 (2)	
C3	0.70323 (11)	0.26362 (10)	0.35548 (5)	0.0225 (2)	
H3	0.6495	0.1886	0.3328	0.027*	
C4	0.80589 (11)	0.24503 (11)	0.41754 (6)	0.0256 (2)	
H4	0.8212	0.1549	0.4361	0.031*	
C5	0.86349 (11)	0.47088 (11)	0.42454 (6)	0.0258 (2)	
H5	0.9188	0.5440	0.4483	0.031*	
C6	0.76529 (11)	0.49972 (10)	0.36234 (6)	0.0236 (2)	
H6	0.7556	0.5902	0.3437	0.028*	
C7	0.35237 (11)	0.35774 (10)	0.18088 (5)	0.0215 (2)	
C8	0.22392 (11)	0.40448 (11)	0.20807 (6)	0.0253 (2)	
C9	0.09606 (12)	0.41663 (12)	0.15761 (6)	0.0308 (3)	
H9	0.0067	0.4474	0.1743	0.037*	
C10	0.09807 (12)	0.38435 (12)	0.08382 (6)	0.0322 (3)	
H10	0.0099	0.3923	0.0504	0.039*	
C11	0.22720 (13)	0.34047 (11)	0.05807 (6)	0.0295 (2)	
H11	0.2270	0.3205	0.0069	0.035*	
C12	0.35778 (12)	0.32520 (10)	0.10616 (6)	0.0247 (2)	
C13	0.49893 (12)	0.26737 (11)	0.08127 (6)	0.0297 (2)	
H13	0.5385	0.1989	0.1193	0.036*	
C14	0.61908 (13)	0.37664 (13)	0.07854 (6)	0.0345 (3)	
H14A	0.5906	0.4386	0.0370	0.052*	
H14B	0.7133	0.3326	0.0719	0.052*	
H14C	0.6301	0.4284	0.1250	0.052*	
C15	0.47312 (15)	0.19216 (13)	0.00699 (7)	0.0406 (3)	
H15A	0.3950	0.1239	0.0089	0.061*	
H15B	0.5648	0.1468	−0.0027	0.061*	
H15C	0.4431	0.2577	−0.0327	0.061*	
C16	0.21896 (12)	0.43226 (12)	0.29009 (6)	0.0307 (3)	
H16	0.3236	0.4405	0.3138	0.037*	
C17	0.14987 (15)	0.31045 (14)	0.32582 (7)	0.0400 (3)	
H17A	0.0468	0.2997	0.3040	0.060*	
H17B	0.1532	0.3261	0.3792	0.060*	
H17C	0.2054	0.2275	0.3172	0.060*	
C18	0.14025 (15)	0.56513 (13)	0.30479 (7)	0.0404 (3)	
H18A	0.1874	0.6410	0.2815	0.061*	
H18B	0.1467	0.5807	0.3583	0.061*	
H18C	0.0360	0.5590	0.2840	0.061*	
N1	0.48109 (9)	0.33206 (9)	0.23309 (5)	0.02100 (19)	
H20	0.4927 (15)	0.2479 (16)	0.2480 (8)	0.034 (3)*	
N2	0.88469 (10)	0.34533 (9)	0.45299 (5)	0.0264 (2)	
O1	0.55121 (8)	0.55210 (7)	0.24345 (4)	0.02682 (19)	

### Atomic displacement parameters (Å^2^)

**Table:**

	*U*^11^	*U*^22^	*U*^33^	*U*^12^	*U*^13^	*U*^23^
C1	0.0212 (5)	0.0163 (5)	0.0221 (5)	0.0015 (4)	0.0004 (4)	−0.0016 (4)
C2	0.0191 (4)	0.0190 (5)	0.0201 (5)	0.0019 (4)	0.0011 (4)	−0.0017 (4)
C3	0.0256 (5)	0.0185 (5)	0.0228 (5)	−0.0001 (4)	0.0000 (4)	−0.0015 (4)
C4	0.0301 (5)	0.0213 (5)	0.0244 (5)	0.0025 (4)	−0.0011 (4)	0.0021 (4)
C5	0.0250 (5)	0.0240 (5)	0.0270 (5)	−0.0004 (4)	−0.0032 (4)	−0.0042 (4)
C6	0.0240 (5)	0.0182 (5)	0.0275 (5)	0.0010 (4)	−0.0015 (4)	−0.0013 (4)
C7	0.0236 (5)	0.0148 (4)	0.0243 (5)	−0.0028 (4)	−0.0043 (4)	0.0012 (4)
C8	0.0244 (5)	0.0222 (5)	0.0281 (5)	−0.0029 (4)	−0.0020 (4)	0.0003 (4)
C9	0.0232 (5)	0.0303 (6)	0.0371 (6)	−0.0009 (4)	−0.0038 (4)	0.0014 (5)
C10	0.0305 (5)	0.0279 (6)	0.0341 (6)	−0.0044 (4)	−0.0126 (4)	0.0042 (4)
C11	0.0404 (6)	0.0224 (5)	0.0231 (5)	−0.0049 (4)	−0.0067 (4)	0.0011 (4)
C12	0.0323 (5)	0.0162 (5)	0.0247 (5)	−0.0032 (4)	−0.0007 (4)	0.0014 (4)
C13	0.0370 (6)	0.0247 (5)	0.0277 (5)	0.0010 (4)	0.0044 (4)	0.0005 (4)
C14	0.0360 (6)	0.0361 (6)	0.0317 (6)	−0.0025 (5)	0.0051 (5)	−0.0008 (5)
C15	0.0519 (7)	0.0347 (7)	0.0369 (6)	−0.0063 (6)	0.0126 (5)	−0.0088 (5)
C16	0.0240 (5)	0.0382 (6)	0.0294 (6)	−0.0007 (4)	0.0007 (4)	−0.0035 (5)
C17	0.0483 (7)	0.0395 (7)	0.0326 (6)	0.0046 (6)	0.0063 (5)	0.0054 (5)
C18	0.0472 (7)	0.0360 (7)	0.0402 (7)	−0.0020 (5)	0.0144 (5)	−0.0038 (5)
N1	0.0231 (4)	0.0146 (4)	0.0236 (4)	−0.0002 (3)	−0.0040 (3)	0.0009 (3)
N2	0.0271 (4)	0.0266 (5)	0.0241 (4)	0.0023 (4)	−0.0030 (3)	−0.0008 (3)
O1	0.0308 (4)	0.0150 (4)	0.0317 (4)	0.0002 (3)	−0.0081 (3)	0.0008 (3)

### Geometric parameters (Å, º)

**Table:**

C1—O1	1.2325 (12)	C11—C12	1.3968 (15)
C1—N1	1.3366 (13)	C11—H11	0.9500
C1—C2	1.5057 (13)	C12—C13	1.5215 (15)
C2—C6	1.3891 (14)	C13—C15	1.5324 (16)
C2—C3	1.3908 (14)	C13—C14	1.5357 (16)
C3—C4	1.3885 (14)	C13—H13	1.0000
C3—H3	0.9500	C14—H14A	0.9800
C4—N2	1.3364 (14)	C14—H14B	0.9800
C4—H4	0.9500	C14—H14C	0.9800
C5—N2	1.3402 (14)	C15—H15A	0.9800
C5—C6	1.3845 (14)	C15—H15B	0.9800
C5—H5	0.9500	C15—H15C	0.9800
C6—H6	0.9500	C16—C18	1.5256 (17)
C7—C8	1.3996 (15)	C16—C17	1.5302 (17)
C7—C12	1.4025 (14)	C16—H16	1.0000
C7—N1	1.4392 (12)	C17—H17A	0.9800
C8—C9	1.3981 (14)	C17—H17B	0.9800
C8—C16	1.5224 (15)	C17—H17C	0.9800
C9—C10	1.3812 (17)	C18—H18A	0.9800
C9—H9	0.9500	C18—H18B	0.9800
C10—C11	1.3844 (17)	C18—H18C	0.9800
C10—H10	0.9500	N1—H20	0.872 (16)
			
O1—C1—N1	122.33 (9)	C15—C13—C14	109.99 (9)
O1—C1—C2	120.52 (9)	C12—C13—H13	107.0
N1—C1—C2	117.12 (8)	C15—C13—H13	107.0
C6—C2—C3	117.96 (9)	C14—C13—H13	107.0
C6—C2—C1	117.57 (9)	C13—C14—H14A	109.5
C3—C2—C1	124.40 (9)	C13—C14—H14B	109.5
C4—C3—C2	118.28 (9)	H14A—C14—H14B	109.5
C4—C3—H3	120.9	C13—C14—H14C	109.5
C2—C3—H3	120.9	H14A—C14—H14C	109.5
N2—C4—C3	124.45 (9)	H14B—C14—H14C	109.5
N2—C4—H4	117.8	C13—C15—H15A	109.5
C3—C4—H4	117.8	C13—C15—H15B	109.5
N2—C5—C6	123.51 (10)	H15A—C15—H15B	109.5
N2—C5—H5	118.2	C13—C15—H15C	109.5
C6—C5—H5	118.2	H15A—C15—H15C	109.5
C5—C6—C2	119.31 (9)	H15B—C15—H15C	109.5
C5—C6—H6	120.3	C8—C16—C18	113.21 (10)
C2—C6—H6	120.3	C8—C16—C17	109.78 (10)
C8—C7—C12	122.98 (9)	C18—C16—C17	111.51 (10)
C8—C7—N1	118.18 (9)	C8—C16—H16	107.4
C12—C7—N1	118.65 (9)	C18—C16—H16	107.4
C9—C8—C7	117.42 (10)	C17—C16—H16	107.4
C9—C8—C16	120.54 (10)	C16—C17—H17A	109.5
C7—C8—C16	121.91 (9)	C16—C17—H17B	109.5
C10—C9—C8	120.78 (10)	H17A—C17—H17B	109.5
C10—C9—H9	119.6	C16—C17—H17C	109.5
C8—C9—H9	119.6	H17A—C17—H17C	109.5
C9—C10—C11	120.68 (10)	H17B—C17—H17C	109.5
C9—C10—H10	119.7	C16—C18—H18A	109.5
C11—C10—H10	119.7	C16—C18—H18B	109.5
C10—C11—C12	120.95 (10)	H18A—C18—H18B	109.5
C10—C11—H11	119.5	C16—C18—H18C	109.5
C12—C11—H11	119.5	H18A—C18—H18C	109.5
C11—C12—C7	117.17 (10)	H18B—C18—H18C	109.5
C11—C12—C13	122.60 (10)	C1—N1—C7	122.32 (8)
C7—C12—C13	120.11 (9)	C1—N1—H20	121.6 (9)
C12—C13—C15	113.31 (10)	C7—N1—H20	115.5 (9)
C12—C13—C14	112.29 (9)	C4—N2—C5	116.45 (9)
			
O1—C1—C2—C6	1.35 (14)	C10—C11—C12—C13	175.36 (10)
N1—C1—C2—C6	−176.83 (9)	C8—C7—C12—C11	−0.33 (15)
O1—C1—C2—C3	178.24 (9)	N1—C7—C12—C11	174.69 (9)
N1—C1—C2—C3	0.05 (14)	C8—C7—C12—C13	−176.56 (9)
C6—C2—C3—C4	1.29 (14)	N1—C7—C12—C13	−1.54 (14)
C1—C2—C3—C4	−175.58 (9)	C11—C12—C13—C15	−16.18 (15)
C2—C3—C4—N2	0.63 (16)	C7—C12—C13—C15	159.84 (10)
N2—C5—C6—C2	1.24 (16)	C11—C12—C13—C14	109.19 (11)
C3—C2—C6—C5	−2.17 (15)	C7—C12—C13—C14	−74.79 (12)
C1—C2—C6—C5	174.92 (9)	C9—C8—C16—C18	−48.05 (14)
C12—C7—C8—C9	0.93 (15)	C7—C8—C16—C18	136.15 (11)
N1—C7—C8—C9	−174.12 (9)	C9—C8—C16—C17	77.27 (13)
C12—C7—C8—C16	176.85 (10)	C7—C8—C16—C17	−98.53 (12)
N1—C7—C8—C16	1.81 (15)	O1—C1—N1—C7	−8.80 (15)
C7—C8—C9—C10	−0.44 (16)	C2—C1—N1—C7	169.34 (8)
C16—C8—C9—C10	−176.43 (10)	C8—C7—N1—C1	−76.45 (12)
C8—C9—C10—C11	−0.62 (17)	C12—C7—N1—C1	108.28 (11)
C9—C10—C11—C12	1.25 (17)	C3—C4—N2—C5	−1.59 (16)
C10—C11—C12—C7	−0.76 (15)	C6—C5—N2—C4	0.63 (15)

### Hydrogen-bond geometry (Å, º)

Cg1 and Cg2 are the centroids of the phenyl and pyridyl rings, respectively.

**Table:**

*D*—H···*A*	*D*—H	H···*A*	*D*···*A*	*D*—H···*A*
N1—H20···O1^i^	0.872 (16)	1.970 (16)	2.7985 (11)	158.3 (13)
C3—H3···O1^i^	0.95	2.54	3.4500 (12)	161
C5—H5···N2^ii^	0.95	2.61	3.4983 (13)	155
C6—H6···*Cg*1^iii^	0.95	2.81	3.6467	150
C15—H15*B*···*Cg*2^iv^	0.98	2.97	3.7858	143

Symmetry codes: (i) −*x*+1, *y*−1/2, −*z*+1/2; (ii) −*x*+2, −*y*+1, −*z*+1; (iii) −*x*+1, *y*+1/2, −*z*+1/2; (iv) *x*, −*y*−1/2, *z*−3/2.
